# Editorial: Exploring chronic fatigue: neural correlates, mechanisms, and therapeutic strategies

**DOI:** 10.3389/fnins.2025.1751667

**Published:** 2025-12-10

**Authors:** Sławomir Kujawski, Lynette Hodges, Karl J. Morten, Paweł Zalewski

**Affiliations:** 1 Department of Exercise Physiology and Functional Anatomy, Ludwik Rydygier Collegium Medicum in Bydgoszcz Nicolaus Copernicus University in Toruń, Bydgoszcz, Poland; 2 School of Sport, Exercise and Nutrition, Massey University, Palmerston North, New Zealand; 3 The Nuffield Department of Women's and Reproductive Health, The University of Oxford, The Women Centre, The John Radcliffe Hospital, Oxford, United Kingdom; 4 Department of Experimental and Clinical Physiology, Laboratory of Centre for Preclinical Research, Warsaw Medical University, Warsaw, Poland

**Keywords:** ME/CFS, brain, autonomic nervous system, central autonomic network, heart rate variability, blood pressure, multiple sclerosis, neurovisceral integration

## 1 Introduction

Fatigue and weariness have been universal experiences throughout human history, coexisting with humanity since its earliest days across all cultures and times. It occurs in ancient stories, including Genesis, in which Adam's fatigue was linked to the toil imposed upon him as part of the consequences of disobedience, a condition that made sustaining life a laborious task. Acute fatigue, which arises naturally in response to stress or work, is a normal physiological process experienced by all humans regardless of era or place. It signals the body's need to rest and adapt, playing a vital role in maintaining health and balance.

In contrast, chronic fatigue, as seen in aging populations and conditions like myalgic encephalomyelitis/chronic fatigue syndrome (ME/CFS), is a complex and often debilitating disorder that extends beyond normal tiredness. It involves sustained disruption of metabolic, neurological, and immune functions, resisting typical recovery mechanisms. The 14 papers in this Research Topic collectively explore the multifaceted nature of fatigue, presenting advances in mechanistic research, epidemiology, clinical interventions, rehabilitation techniques, and innovative monitoring technologies aimed at improving diagnosis, treatment, and management of this persistent condition.

## 2 Epidemiology and public health perspectives

### 2.1 Fatigue prevalence in older adults in Ethiopia

Mekuria et al. address fatigue's burden in an underserved population by conducting a cross-sectional survey of older adults in Bahir Dar, Ethiopia. Finding a high fatigue prevalence (~38%), the study associates fatigue with advanced age, increased comorbidities, physical inactivity, poor social support, insomnia, and depression, illustrating the psychosocial and medical complexity underlying fatigue in aging low-income contexts.

### 2.2 Navigating the pharmacological landscape of ME/CFS in women: a call for precision medicine

This study offers a timely and nuanced look into the pharmacological landscape of ME/CFS among North American women, revealing a high reliance on analgesics and psychotropics despite limited evidence for their efficacy. The U-shaped relationship between medication use and physical activity underscores the delicate balance ME/CFS patients must strike to avoid post-exertional malaise. As no curative treatments exist, these findings highlight the urgent need for personalized, evidence-based therapeutic strategies that address the condition's heterogeneity, moving beyond trial-and-error toward targeted, mechanism-informed care that truly improves function and quality of life (Pochakom et al.).

## 3 Mechanistic foundations of physiology and pathology of fatigue in humans and in animal models

### 3.1 Integrated animal model of central fatigue

Zhang et al. pioneered a rat model of central fatigue that uniquely integrates sleep disruption, a high-fat diet (HFD), and alternate-day fasting (ADF). The study demonstrates that while HFD alone is insufficient to induce central fatigue, the combination of HFD with ADF, paired with sleep deprivation, synergistically triggers robust fatigue phenotypes. These include reduced locomotor activity, impaired exercise endurance, cognitive deficits, hippocampal neurodegeneration, mitochondrial ultrastructural damage, and elevated oxidative stress.

### 3.2 Machine learning classification of pilot fatigue

Guo et al. harness machine learning algorithms to classify pilot fatigue states based on physiological features, including heart rate variability and respiratory patterns collected during actual flights. Their tree-based classification model achieves high accuracy, paving the way for real-time fatigue monitoring in high-stakes operational environments. This innovation demonstrates how predictive analytics can augment human performance and safety monitoring in demanding professions.

### 3.3 Uncovering a structural signature of chronic fatigue syndrome

This study leverages machine learning and structural MRI to uncover cortical atrophy linked to CFS, identifying five key brain regions, spanning motor, parietal, and temporal cortices. Reduced thickness in these areas was found to correlate with greater symptom severity, increased pain, and diminished quality of life. The authors offer a promising structural biomarker framework for CFS, a condition long hindered by diagnostic ambiguity. While limited by sample size and cross-sectional design, the work advances neurobiological understanding of CFS and underscores the potential of cortical morphology in clinical assessment and future therapeutic targeting (Wu et al.).

### 3.4 Central sensitization and nociplastic mechanisms in chronic fatigue

Chen et al. propose that myalgic encephalomyelitis/chronic fatigue syndrome (ME/CFS) and Gulf War Illness (GWI) involve central sensitization driven by nociplastic and interoplastic mechanisms, resulting in amplified pain and interoceptive symptoms. Using dolorimetry, they found objective evidence of systemic hyperalgesia that correlates strongly with pain and interoception but only weakly with fatigue or disability. In contrast, Chronic Idiopathic Fatigue (CIF) exhibited intermediate tenderness and lacked the full nociplastic/interoplastic profile, suggesting it may represent a distinct clinical entity. These findings call for further research into the neural substrates underlying these symptom dimensions.

### 3.5 Metabolomic and proteomic dysregulation in chronic fatigue syndromes

Davis et al. present an integrative review of metabolomic and proteomic profiles across ME/CFS, Gulf War Illness, and fibromyalgia. Common metabolic disruptions emerge, including dysregulated lipid metabolism, mitochondrial energy deficits, heightened oxidative stress, and persistent inflammation. These molecular patterns underpin shared pathophysiology across diverse fatigue syndromes, suggesting that targeting these core biochemical pathways may yield broad-spectrum therapeutic benefits and improve biomarker-guided diagnoses.

## 4 Intervention-based studies, meta-analysis, cross-sectional study, and protocol

### 4.1 Oxaloacetate supplementation reduces fatigue and improves cognitive function in ME/CFS (RESTORE ME Trial)

Cash et al. randomized controlled trial (RCT) (RESTORE ME Trial) assessed oxaloacetate supplementation in ME/CFS patients. Over 3 months, oxaloacetate significantly reduced fatigue, likely by enhancing mitochondrial metabolic pathways and reducing neuroinflammation. Additionally, Vernon, Rond, Sun, et al. found that oxaloacetate may improve cognitive function and slightly increase upright activity in people with ME/CFS, revealing a strong link between fatigue and cognition within the oxaloacetate group that suggests a treatable subgroup. Although the study employed objective measures such as digital cognitive testing and wearable activity tracking, its modest sample size and 90-day duration limit definitive conclusions. Despite these promising results, further RCTs are needed to replicate existing therapies' findings and confirm oxaloacetate's effects, as such studies remain scarce in ME/CFS research.

### 4.2 Oxaloacetate supplementation might improve cognitive performance in long COVID (REGAIN trial)

The REGAIN trial offers a nuanced perspective on treating long COVID, revealing that while oxaloacetate did not significantly reduce fatigue on the Chalder scale, it did drive earlier improvements in overall symptom burden and cognitive function. Participants receiving oxaloacetate demonstrated improvement in some of the examined cognitive function indicators. Though larger and longer studies are needed, these findings suggest oxaloacetate may engage neurometabolic pathways critical to long COVID recovery, warranting serious consideration as a therapeutic strategy, after results are replicated in a RCT involving a bigger sample size (Vernon, Rond, Bell, et al.).

### 4.3 Muscle fatigue assessment and autonomic dysfunction in ME/CFS

Schlömer et al. combine clinical muscle fatigue metrics using handgrip strength with assessments of autonomic dysfunction via passive standing tests in ME/CFS. They report that pyridostigmine, a cholinesterase inhibitor, markedly enhances muscle recovery and reduces orthostatic heart rate increases. These findings offer a clinically actionable approach to multifaceted fatigue management in ME/CFS.

### 4.4 Fatigue, respiratory function, and core morphology in multiple sclerosis

De La Plaza San Frutos et al. investigate fatigue determinants in multiple sclerosis (MS), linking fatigue severity to respiratory muscle weakness and altered core muscle morphology observed via ultrasound. Their observational data suggest that restoring respiratory muscle function and core stability could alleviate balance issues and reduce fatigue, expanding therapeutic considerations in MS rehabilitation beyond conventional neurological management. Further studies are needed to explore the indirect effects of pleiotropic therapies, including different types of physical exercise programs, in MS.

### 4.5 Meta-analysis on the effectiveness of respiratory muscle training in multiple sclerosis

This systematic review and meta-analysis by Xiang et al. offers timely, clinically relevant insights into respiratory muscle training for patients with MS. While this intervention significantly improves inspiratory and expiratory muscle strength and reduces fatigue, its effects on lung function, exercise capacity, and quality of life remain uncertain, likely due to small, heterogeneous studies with methodological limitations. The findings underscore the need for larger, rigorously designed randomized trials, particularly in those with a greater Expanded Disability Status Score (EDSS) score. Nonetheless, respiratory muscle training emerges as a promising, low-risk adjunct in MS rehabilitation, warranting individualized implementation based on disability level and symptom profile.

### 4.6 Foam rolling vs. trunk stabilization for muscle fatigue recovery

Amiri and Zemková focus on mitigating muscle fatigue-related impairments prevalent in sedentary individuals through a randomized clinical trial protocol comparing foam rolling to trunk stabilization exercises. The interventions aim to restore core muscle function and improve postural stability, helping reverse the physical deconditioning and fatigue often arising from prolonged inactivity in sedentary adults. By testing these accessible rehabilitation strategies, this work addresses a common preventable contributor to fatigue and falls risk.

Together, these studies construct a multidimensional framework for fatigue research encompassing mechanistic exploration, population epidemiology, clinical interventions, disease-specific investigations, psychosocial contexts, and innovative technology applications. Their integration from bench to bedside and real-world settings promises to enhance diagnostics, treatments, and preventive strategies, addressing fatigue's pervasive burden across medical and occupational domains.

